# Antimicrobial Activities of Marine Sponge-Associated Bacteria

**DOI:** 10.3390/microorganisms9010171

**Published:** 2021-01-14

**Authors:** Yitayal S. Anteneh, Qi Yang, Melissa H. Brown, Christopher M. M. Franco

**Affiliations:** 1 College of Medicine and Public Health, Flinders University, Bedford Park, SA 5042, Australia; yitayal.shiferaw@aau.edu.et; 2 Department of Medical Microbiology, College of Medicine, Addis Ababa University, Addis Ababa 9086, Ethiopia; 3 Center for Marine Drugs, State Key Laboratory of Oncogene and Related Genes, Department of Pharmacy, Renji Hospital, School of Medicine, Shanghai Jiao Tong University, Shanghai 200127, China; qi.yang@flinders.edu.au; 4 Centre for Marine Bioproducts Development, College of Medicine and Public Health, Flinders University, Bedford Park, SA 5042, Australia; 5 College of Science and Engineering, Flinders University, Bedford Park, SA 5042, Australia; melissa.brown@flinders.edu.au

**Keywords:** marine sponges, Actinobacteria, antimicrobial, dermatophytes, South Australia

## Abstract

The misuse and overuse of antibiotics have led to the emergence of multidrug-resistant microorganisms, which decreases the chance of treating those infected with existing antibiotics. This resistance calls for the search of new antimicrobials from prolific producers of novel natural products including marine sponges. Many of the novel active compounds reported from sponges have originated from their microbial symbionts. Therefore, this study aims to screen for bioactive metabolites from bacteria isolated from sponges. Twelve sponge samples were collected from South Australian marine environments and grown on seven isolation media under four incubation conditions; a total of 1234 bacterial isolates were obtained. Of these, 169 bacteria were tested in media optimized for production of antimicrobial metabolites and screened against eleven human pathogens. Seventy bacteria were found to be active against at least one test bacterial or fungal pathogen, while 37% of the tested bacteria showed activity against *Staphylococcus aureus* including methicillin-resistant strains and antifungal activity was produced by 21% the isolates. A potential novel active compound was purified possessing inhibitory activity against *S. aureus.* Using 16S rRNA, the strain was identified as *Streptomyces* sp. Our study highlights that the marine sponges of South Australia are a rich source of abundant and diverse bacteria producing metabolites with antimicrobial activities against human pathogenic bacteria and fungi.

## 1. Introduction

Excessive and misuse of antimicrobial agents has been attributed to the emergence of multidrug-resistant (MDR) microorganisms, which increases the mortality rate associated with infectious diseases [1,2]. This multidrug resistance and associated mortality demand a redoubling of efforts to find new effective antimicrobials from alternative sources. In the last decade this has included the development of novel antimicrobials from different nanocompounds [3,4]. However, as approximately 50% of the new drugs in the antibiotic pipeline have originated from natural products, it was decided to survey marine microorganisms [5]. The marine environment is an abundant source of active natural compounds with biological and pharmaceutical properties [6,7]. Each year, the amount of metabolically-active compounds from the marine environment increases and sponges contribute a large share with about 200 new active compounds identified annually where the nature of active compounds varies depending on the sponges species [8,9,10]. Due to the nature of marine environments compared with their terrestrial counterparts, marine sponges may be exposed to different challenges which stimulate production of biologically-active molecules as defense mechanisms [10]. These active metabolites from marine sponges isolated from various geographical locations can be nucleoside derivatives, terpenoids, polyethers, alkaloids, macrolides, or peptides with various biological activities including anti-inflammatory, anti-cancer and antimicrobial properties [10,11,12,13,14,15].

Marine sponges (phylum Porifera) are one of the earliest life forms that exist in the world (~630 million years) [16]. They are the second largest benthic community compared to coral reefs, and the diversity of sponge species outnumbers the combined species diversity of other organisms in the community [17]. The mesohyl of many sponges is heavily populated by microbial symbionts, including bacteria, fungi, viruses, and archaea, which account for up to 50% of sponge biomass in the case of high microbially abundant sponges [18]. Microbes surviving in these extremely nutrient-poor and antagonistic environments, such as marine sponges, frequently produce a variety of secondary metabolites to overcome the negative effect of the surrounding environment [19,20]. Due to the requirement for large amounts of sponge biomass for the production of any bioactive and mass cultivation of marine sponges being difficult, the utilization of sponges as a source of antibiotics is hindered [21]. The observed structural similarities among active compounds from marine sponges and terrestrial microorganisms indicated that sponge associated bacteria could be the main sources of some of these active compounds [22,23,24,25]. Moreover, numerous studies have identified a wide range of antimicrobial activities from sponge-associated microbes, which makes these microbial populations an important source for novel antimicrobials [26,27,28].

The phylum Actinobacteria contributes to the major share of active metabolites from sponges [29]. A comprehensive study revealed the presence of various Actinobacteria with a range of antimicrobial activities [30] and others have also reviewed metabolites with different biological activities from sponge-associated culturable and unculturable microbial population [21,25,31]. Clinically-significant bioactive compounds from marine sponge-associated microbes have been reported from different geographical areas including the Great Barrier Reef of Australia, South China Sea, Mediterranean Sea, Indonesia, Papua New Guinea, and the Indo-Pacific region, to name a few [31,32]. Antimicrobials, such as pyrrolo[1,2-a] pyrazine-1,4-dione, hexahydro [33], naphthacene glycoside [34], kocurin [35], and saadamycin [36], were among the many compounds obtained from marine sponge-associated bacteria.

Cultivation of sponges at an industrial scale for mass production of active compounds has not yet been achieved though there have been recent attempts to use resin to capture sponge metabolites from sponge kept alive in an aquarium [37]. On the other hand, if compounds are produced by sponge-associated microbes, the scale-up is relatively easy to achieve.

Australia completely separated from Antarctica more than 40 million years ago to form a continent with a large latitude span with different climatic zones, which contribute to the greater biodiversity of flora and fauna on land and sea. Thus, Australia, particularly the Southern region, is a source of huge diversity of endemic sponge species [38]. Sorokin et al., (2007) and Sorokin & Currie (2008) observed a significant variation in the chemical composition among sponges of South Australia, which forms the basis to hypothesize the associated symbionts and/or the metabolites from them are different [39,40]. Therefore, this study aimed to isolate sponge-associated bacteria from marine sponges of South Australia and screen their metabolites for antimicrobial activities. The study also investigated the effect of media and incubation time on the production of antimicrobial compounds from bacteria and attempted to characterize potential novel antibiotics.

## 2. Materials and Methods

### 2.1. Sponge Collection, Processing, and Identification

Sponges in this study were sampled under Exemption Permit Number 9,902,620 by the South Australian Research Development Institute (SARDI), issued by Primary Industries and Regions South Australia. Materials were used by Flinders University under a Material Transfer Agreement with SARDI and did not involve endangered or protected species. Twelve sponge samples were collected from South Australian marine environments by scuba diving. Four samples were from Glenelg blocks (34°58′406″ S, 138°30′494″ E) assigned the sampling codes of GB_SP_01, 08, 21, and 23. The rest eight were from Rapid Bay Jetty (35°31′39″ S 138°11′22″ E) with the codes of RB_SP_01-03, 11, 12, 16–18. The samples were collected in sterile Ziploc plastic bags containing fresh seawater and transported to the laboratory in an icebox. All samples were washed in sterilized seawater to remove any external organic matter followed by surface sterilization with 70% ethanol. All the samples were dried in a sterile laminar flow chamber. The samples were cut into small pieces with size of approximately 1 cm^3^ for different analyses: (1) sponge dilution preparation for microbial isolation, (2) storage in 70% ethanol for sponge morphological identification, and (3) frozen in −80 °C freezer for DNA extraction.

Sponge samples were firstly classified based on morphological characterization [41,42] and the spicule preparation have followed the methods in ‘sponguide’ [43]. In addition, ambiguous samples were further distinguished and identified using 28S rRNA gene following a previously described protocol [44]. Briefly, the frozen sponge samples were crushed in liquid nitrogen and their tissue lysed with cetyltrimethylammonium bromide (CTAB) extraction buffer [45]. The mix was then combined with polyvinylpyrrolidone and β-mercaptoethanol to help to remove tannins and phenolic compounds [46]. The addition of phenol: chloroform: isoamyl allowed the separation of nucleic acids from proteins and polysaccharides, and finally the DNA was precipitated with iced isopropanol and reconstituted with 50 µl injection water and stored at −20 °C. The quality and the quantity of the DNA was assessed with a Nanodrop 1000 Spectrophotometer (Thermo Scientific, Wilmington, DE, USA) and those with high quality was considered for PCR reactions. D3- D5 regions of the 28S rRNA gene of sponge DNA were amplified by the primer set NL4F (5′-GAC-CCG-AAA-GAT-GGT-GAA-CTA-3,) and NL4R (5′-ACC-TTG-GAG-ACC-TGA-TGC-G-3′) [44]. Thermocycler conditions were as follows: a 10-min initial denaturation at 95 °C; 35 cycles of 95 °C for 1 min, 56 °C for 1 min and 72 °C for 1 min; and a final extension step at 72 °C for 7 min. The PCR products were cleaned and sent for sequencing to Macrogen, South Korea. Identities were inferred by comparing the sequence using BLAST against the NCBI database. The qualified sequences used to infer the taxonomic identities have been submitted to NCBI GenBank and assigned with a unique Accession number of MN310319-MN310366, MN315546- MN315548, MN315519-MN315531, MK367393, MK367392, and MK358953.

### 2.2. Isolation of Bacteria

One piece of dried and surface sterilized sponge (approximately 1 cm^3^) was cut from the inner mesohyl and ground in 10 volumes of sterile seawater using a clean, sterile pestle and mortar. A 10-fold dilution series (10^−1^ to 10^−6^) was prepared and 100 µL of the three highest dilutions were inoculated onto different primary isolation media, which are selected from previous studies, in six replicates.

Seven different media SYP [47], asparagine peptone agar [48], natural seawater agar (NS) [47], humic acid vitamin agar [49], marine agar [47], nutrient agar [50] and tryptone soya agar [50] were used for bacterial isolations. Plates were incubated at 15 °C and 27 °C at aerobic, microaerophilic, and anaerobic conditions over 16 weeks to ensure for the isolation of less abundant bacteria as they require longer period to form colonies. Once a week, colonies were picked out from primary isolation media [49] and sub-cultured onto SYP, ISP2 and NA plates to get single pure colonies. Pure cultures were stored on plates and agar slants for short periods, and colonies were also placed in 30% glycerol and stored at −80 °C for future use [51].

### 2.3. Morphological Identification of Bacteria

All bacterial isolates were sub-cultured onto three identification media (SYP, ISP2 and NA) and categorized based on the similarity of their colony morphology; namely colony colour and nature of the spores. Microscopic observation of stained (Gram staining) and unstained (wet mount) bacteria was further applied for identification. Bacteria identification was done with two skilled personnel to reduce the observer bias.

### 2.4. Genotypic Identification of Bacteria

Genomic DNA of all bacterial isolates was extracted using a CTAB method as described previously [52]. Two sets of primers (27F (5′-GAG-AGT-TTG-ATC-CTG-GCT-CAG-3′), 765R (5′-CTG-TTT-GCT-CCC-CAC-GCT-TTC-3′)) and (704F (5′-GTA-GCG-GTG-AAA-TGC-GTA-GA-3′), 1492R (5′-CAC-GGA-TCC-TAC-GGG-TAC-CTT-GTT-ACG-ACT-T-3′)) were used for amplification of the bacterial 16S rRNA gene. Amplified products were digested with *Hha*l *and Pst*I restriction enzymes for RFLP-based categorization of the isolates. Representative PCR products from each RFLP pattern were sequenced and the NCBI database was used for BLASTN analysis. Sequences were initially aligned using the multiple alignment program CLUSTAL W version 2.0 [53]. Phylogenetic trees were reconstructed using the neighbor-joining method (based on 1000 bootstrap iterations) with the MEGA version 7 software program [54].

### 2.5. Test Organisms

Bacterial strains that were used for antibacterial screening included *E. coli* JM 109, *P. aeruginosa* 06348315, *S. aureus* ATCC 29213, *S. pyogenes*, *S. typhimurium* and MRSA 03120385. Fungal strains of *C. albicans*, *T. rubrum*, *T. interdigitalis*, *Sacclophosis* sp., and *M. gypsum* were also included as test organisms. All bacteria and *C. albicans* strains were obtained from the Clinical Microbiology and Infectious Diseases Department, Flinders University and the four fungal species were provided by SA pathology, South Australia.

### 2.6. Agar Plugs Antimicrobial Sceening

One hundred and sixty-nine bacterial isolates were selected to represent proportionally different sources, morphology and genotypic patterns, and were sub-cultured in duplicate onto ISP2 medium, for Actinobacterial strains (111 in number) [55] and brain heart infusion agar for other bacterial strains [56] for antimicrobial production [57,58]. Plates were incubated for either 15 (Actinobacterial) or 5 days (for other bacterial strains) at 27 °C in an aerobic environment for optimum antimicrobial production. After incubation, 6-mm plugs from each bacterium were cut out and screened for activities. The other plate was used to obtain an extract. Here, the full plate containing the culture and the agar medium was chopped and placed into a tube containing 40 mL methanol. The tube was shaken for 6 h and the contents centrifuged at 3000 rpm for 10 min. The supernatant was collected and screened for the antimicrobial activity to confirm those strains which showed activities in agar plug assay.

#### 2.6.1. Antibacterial and Anticandida Screening

An agar-based disk diffusion method was used for antibacterial screening. Here ready-made antibiotic assay medium (AAM: Oxoid) was used for the antibacterial activity assay. Test organisms were grown in tryptone soy broth (TSB: Oxoid) at 37 °C by shaking at 150 rpm overnight. Growth was assessed by measuring optical density (OD) at 600 nm and an OD of 0.25 of test organisms were added to the AAM at the ratio of 1 mL per 25 mL of the medium, which was then poured into a 9 cm Petri plate. After drying, a 6 mm plug of 14 (Actinobacteria) or 5-day old (other bacteria) culture was placed onto the test strain (10 plugs per plate). To confirm the activity shown by the cultures on the plug assay, the methanol extract from the agar cultures was also tested in duplicate by being placed into a 6 mm diameter well. Methanol was used as a negative control, with vancomycin (200 µg/mL) and ciprofloxacin (200 µg/mL) as positive controls for Gram-positive and Gram-negative bacteria, respectively. The culture was incubated overnight at 37 °C and the diameter of the inhibition zone was measured in mm. A similar approach was followed for the screening of anti-Candida activities with Sabouraud broth and Sabouraud agar (Oxoid) used for seed medium and antibiotic assays, respectively. Amphotericin B (100 U) was used as positive control and methanol as a negative control. All tests were carried out in duplicate.

#### 2.6.2. Antifungal Screening

Four pathogenic dermatophytic fungal species—*T. rubrum*, *T. interdigitalis*, *Sacclophosis* sp., and *M. gypsum* were grown on potato dextrose agar (Oxoid) at pH 6 for seven days. At the same time, a heavy inoculum of bacteria was streaked on one side of the plate (maximum of three bacteria per plate) and incubated until good growth was observed. A 6 mm plug of test fungi (one per plate) was cut out and placed in the center of the plate (perpendicular to each bacterium streak) and incubated for two weeks. Similarly, a plug of fungus was placed onto the medium without bacteria as a negative control. Results were recorded as follows: very strong inhibition (+4), when the growth of the fungi away from the streaking was >20 mm; strong inhibition (+3), when the growth of the fungi away from the streaking was 15–20 mm; moderate inhibition (+2), at 10–14 mm or less; weak (+1); 10 mm or less; and negative (-), no difference compared to negative control.

### 2.7. Evaluation of Different Media for Antimicrobial Production

Small scale experiment was designed to evaluate primary screening media for initial screening of antimicrobials. Here, we selected eight highly active bacterial strains from the above experiment and their antimicrobial production was evaluated on five common growth/production media: YEA, potato dextrose agar, SYP, mannitol soybean agar (MS) and ISP2. Each strain was inoculated in the same manner using the method mentioned in Section 2.6 and 50 µL of the methanol extracts were screened for antibiotic activities against S. *aureus* and MRSA and the mean zone of inhibition was recorded.

### 2.8. Liquid State Antimicrobial Production, Screening and Large Scale Production

Once the active strains were identified by the agar-plugs screening method, they were screened using different liquid media for mass production of antimicrobials. For this, three highly active bacterial strains (RB27, RB53 and RB154) were selected and processed as follows: two loopfuls of each strain were inoculated into 50 mL of IM22 seed medium (g/L: glucose-15, soyatone-15, pharmamedia-5, CaCO_3_-2 and NaCl- 5) in 250 mL baffled Erlenmeyer flasks and incubated at 27 °C 150 rpm for three days. This initial step helps to standardize the amount of inoculum for the subsequent antimicrobial production. The well-grown seed medium (2.5 mL) was inoculated into 50 mL of four types of liquid production media: (1) sucrose medium (SM (per l; sucrose 20 g, CaCO_3_ 2.5 g, KNO_3_ 1 g, MgSO_4_.7H_2_O 0.5 g, NaCl 0.5 g); (2) ISP2 (malt extract 10 g, yeast extract 4 g, glucose 4 g); (3) glucose medium (GM; (glucose 20 g, soybean flour 10 g, CaCO_3_ 4 g, COCl_2_.6H_2_O 1 mg), and (4) MS (mannitol 20 g, soybean flour 20 g) in 250 mL baffled Erlenmeyer flasks and incubated at 27 °C in shaker of 150 rpm for 7 days. Every day, 1 mL of the culture was collected, centrifuged at 3000 rpm for 10 min and the supernatant screened for antimicrobial activities by disk diffusion method.

Experiment Section 2.8 revealed strain RB 27 produced highly active antimicrobial in MS medium compared to the other strains. As a result, we repeated the same protocol Section 2.8 for production of 5 L of antimicrobials from RB27 using MS medium. The water portion was evaporated off in a tray in a fume hood and the dried powder was stored at −20 °C for further downstream processing.

### 2.9. Bioautogram of Extract from RB27

Fifty microliters of extract dissolved in water was spotted onto a silica gel 60 F_254_ TLC plates (Merck) and placed in a TLC tank containing different eluting solvent systems, including butanol, chloroform:methanol:water (7:7:3); chloroform: methanol (7:7); and pyridine:6MHCl:RO water: methanol (10:4:26:80), and run until the solvent reached 1 cm from the top of the TLC plates. After drying, the plates were observed at 254 and 365 nm and the position of the different compounds marked with a pencil. The area of the TLC plates which contained the compound was cut out and placed onto a medium containing the test organisms as described in Section 2.6.1. The TLC plates were left on the agar medium for 1 h to allow the compound to diffuse into the plate and then removed from the medium. Finally, the plates were incubated overnight at 37 °C and the medium was investigated for the zone of inhibition.

### 2.10. Purfication of Extract from RB27

Data from the preliminary investigation and bioautogram indicated the active compound from strain RB27 is very polar and would not dissolve in methanol. This water-soluble antibiotic was purified with sequential extraction using ethylacetate and water-saturated butanol. Then, once the residual solvent evaporated off, the water-soluble portion was passed through an amberlite XAD-4 column (Amberlite^®^, Sigma-Aldrich, Ausralia) and only the spent was active.

The spent was freeze-dried to remove all liquid and then washed with methanol to remove any solvent-soluble material. In the final step, an off-white powder was dissolved in water and passed through a thin long LH20 (Sephadex), molecular sieve, which separate sample components. Water was used as eluent to remove components from LH20 and the fractions were collected with multiple tubes with 1 mL volume, and each fraction was tested for activity against *S. aureus*. Finally, the active fractions were pooled, freeze-dried and stored at −20 °C until further experimentation.

### 2.11. Nuclear Magnetic Resonance Spectroscopy and Accurate Mass Analysis of Extract from RB27

Forty milligrams of purified extract from strain RB27 were submitted to Flinders University Analytical Centre and Chemistry laboratory (Flinders University) for ^1^H NMR analysis and accurate mass determination. For NMR analysis 20 mg of the extract were dissolved in 500 µL of Deuterated water (D_2_O). NMR analyses were performed using a Bruker 600 MHz spectrometer, equipped with 5 mm inverse multinuclear probe, 5 mm triple resonance probe, variable temperature, z-gradients and autosampler which is optimized for ^1^H and ^13^C detection. ^1^H NMR spectra were acquired at 400 MHz. Standard Bruker software was used to execute recording of 1 and 2 dimensional. All resonance bands were referenced to a tetramethylsilane internal standard [59]. For accurate mass analysis, about 1 mg of the extract was dissolved in 15 mL of acetonitrile and subjected to mass analysis with high-definition mass spectrometer (HDMS).

### 2.12. MIC and MBC of the Compound

For antimicrobial testing, the concentrations of the antimicrobial compound in Mueller Hinton broth ranged from 10 mg/mL to 0.0391 mg/mL. Into each concentration, 100 µL of 1 × 10^7^ CFU/mL of the bacterium was added, and the tubes incubated for 18 h at 37 °C. The MIC was the last dilution with no visible growth of bacterium as indicated by the absence of turbidity in the medium. To obtain the MBC, 10 µL of 1:10 diluted sample from all tubes without visible turbidity were inoculated as a 10 uL drop onto TSA medium and the cultures incubated overnight at 37 °C. The lowest concentration which resulted in a decrease in three logs of growth was reported as the MBC.

## 3. Results

### 3.1. Sponge Taxonomy

Based on morphological characterization, seven of the twelve sponge sampled were identified. GB_SP_01 belongs to *Geodia* sp.; GB_SP_08, 21, and 23 belong to *Chondrosida* sp.; and RB_SP_01, 02 and 03 belong to *Ircinia* sp., *Poecilosclerida* sp. and *Crella* sp., respectively. Using the 28S rRNA gene locus, another five sponge samples were identified as *Sarcotragus* sp. (RB_SP_11) with the (Accession No. EF646841), *Carteriospongia foliascens* (RB_SP_12) (Accession No. KC869574), *Aplysilla sulfurea* (RB_SP_16) (Accession No. EF646837), *Dendrilla* sp. (RB_SP_17) (Accession No. KU533858), and *Tedania tubulifera* (RB_SP_18) (Accession No. KJ620377) [60,61].

### 3.2. Bacterial Profile and Test Strains

A total of 1234 colony-forming bacteria were isolated from the 12 sponge samples. The bacterial isolates were coded with prefixes RB (924 colony-forming units [CFU] and GB (310 CFU) to reflect the sponge collection sites of Rapid Bay and Glenelg Blocks, respectively. These bacteria microscopically appeared as Gram-positive (637), Gram-negative (222), and filamentous bacteria (375). The redundant colonies were further identified and removed based on morphological similarity on three media and microscopy characteristics. A total of 383 morphologically and microscopically different bacterial isolates were selected for further analysis. Digestion of the amplified partial 16S rRNA genes of these 383 bacterial groups resulted in 38 restriction fragment length polymorphism (RFLP) patterns, and the similarity of each pattern was confirmed with morphology and microscopy. Further, 16S rRNA gene sequencing of some of the representative isolates chosen at random from each pattern categorized the bacteria into 21 genera under four phyla of Actinobacteria, Firmicutes, Proteobacteria, and Bacteroidetes. Based on this, there were *Streptomyces* (*n* = 111), *Bacillus* (*n* = 91), *Sulfitobacter* (*n* = 32), *Kocuria* (*n* = 31), *Microbacterium* (*n* = 17), *Limimaricola* (*n* = 17), *Micrococcus* (*n* = 15), *Rhodococcus* (*n* = 8), *Fictibacillus* (*n* = 7), *Gordonia* (*n* = 7), *Pseudonocardia* (*n* = 7), *Flasibacillus* (*n* = 6), *Pseudomonas* (*n* = 6), *Staphylococcus* (*n* = 5), *Rhodovulum* (*n* = 5), *Isoptericola* (*n* = 5), *Leisingera* (*n* = 5), *Mycolicibacterium* (*n* = 3), *Muricauda* (*n* = 3), *Janibacter* (*n* = 1), and *Pseudoalteromonas* (*n* = 1).

One hundred and sixty-nine bacterial strains were selected proportionally from each sponge source and isolation medium (Table 1) for screening for antimicrobial activity against representative Gram-negative bacteria: *Salmonella typhimurium*, *Pseudomonas aeruginosa,* and *Escherichia coli*; Gram-positive bacteria: *Streptococcus pyogenes*, *Staphylococcus aureus* and methicillin-resistant *S. aureus* (MRSA); *Candida albicans* and dermatophytic fungi: *Trichophyton rubrum*, *Trichophyton* *interdigitalis*, *Sacclophosis* sp. and *Microsporum gypsum*. The selection also considered representativeness for morphology, microscopy and genotypic patterns. Specifically, 36 bacterial isolates from Glenelg Blocks and 133 bacterial isolates from Rapid Bay were screened for activities (Supplementary Table S1).

### 3.3. Antimicrobial Activities

About 41% of the isolated bacterial strains produced antimicrobial activities against at least one of the test strains but none of them showed activity against any of the Gram-negative bacterial species or *S. pyogenes* (Supplementary Table S2). The majority (95.7%) of the bacterial strains producing bioactive metabolites were isolated from sponge samples in Rapid Bay. As indicated in Table 2, strains in 10 of 21 genera displayed antimicrobial activities. Six of the 10 active genera belong to phylum Actinobacteria and the other two in phylum Firmicutes and Proteobacteria, respectively (Table 2).

**Figure 1:**
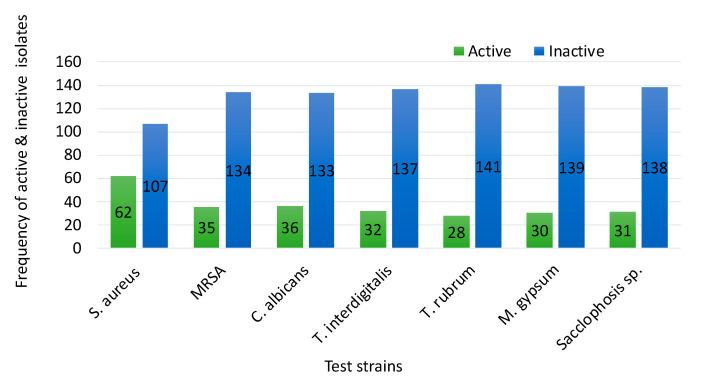
Range of antimicrobial activities of 169 bacterial strains against seven bacterial and fungal pathogens.

As indicated in Figure 1, approximately 37% and 21% of the bacteria were active against *S. aureus* and MRSA, respectively, whereas 27% were active against *C. albicans* and 23% against dermatophytic fungi. Of the 70 bacterial strains with antimicrobial activities, only 10% (seven bacteria) showed activity against a single pathogen, whereas most of them displayed wider ranges of inhibitory activity ranging from two test microorganisms (22 strains) to all of the test organisms (seven strains).

**Figure 2:**
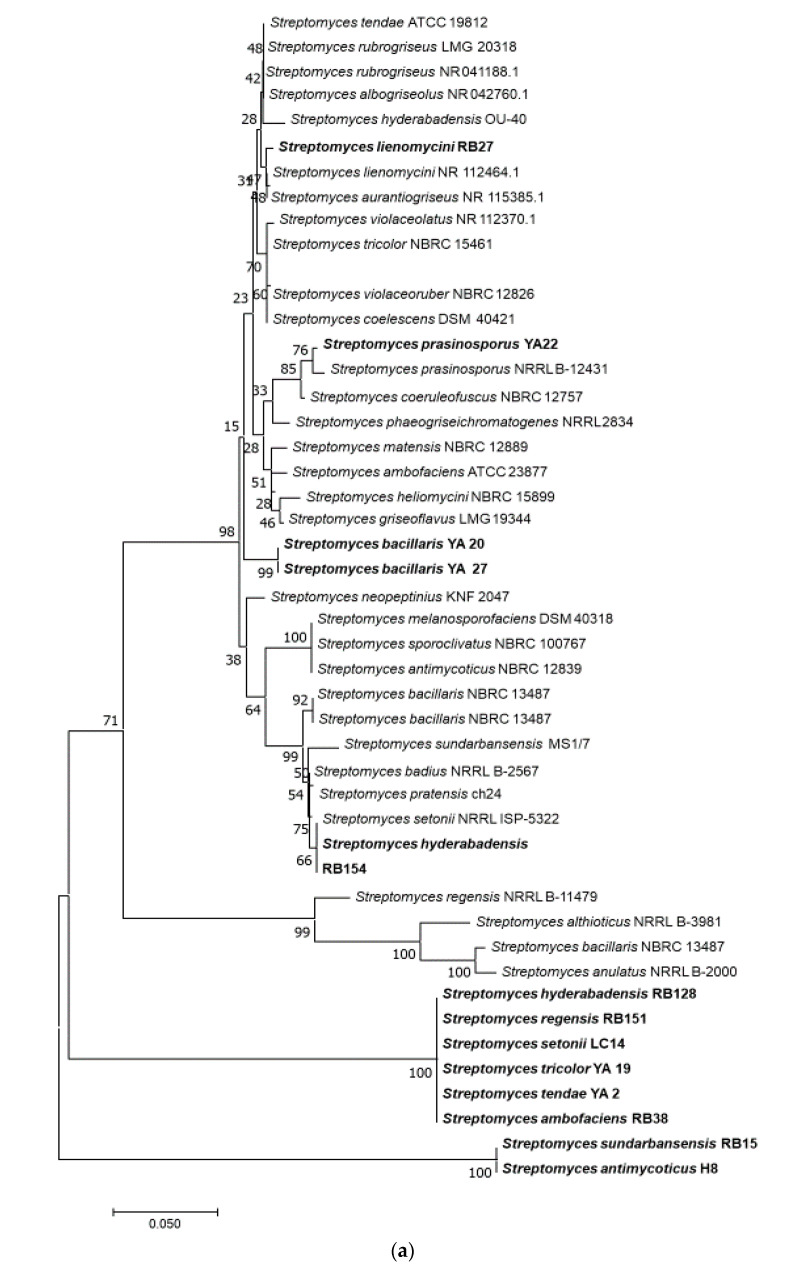

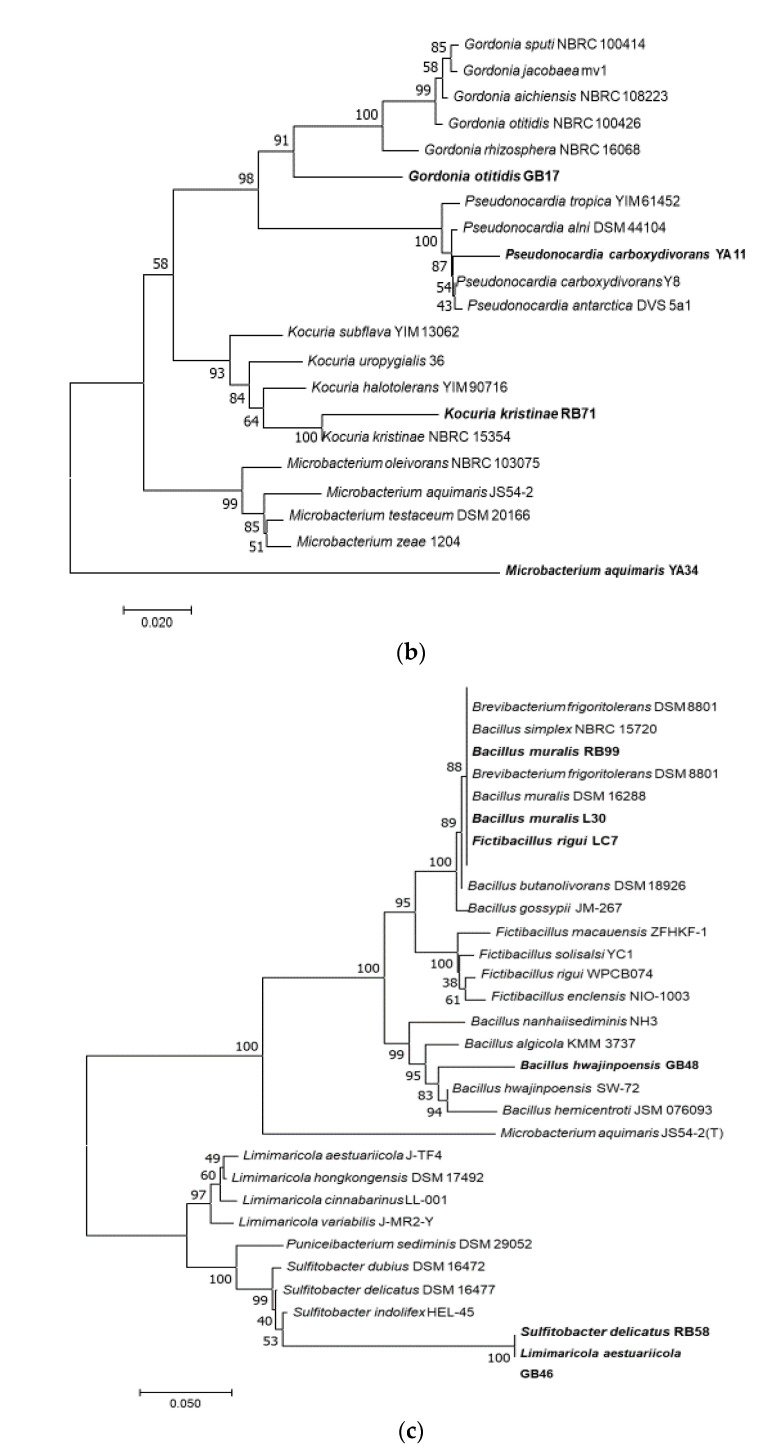
Phylogenetic trees of bacterial strains with demonstrated antimicrobial activities. Neighbour-joining trees shows the relationship between representative sequence(s) from each active bacterial genus. The numbers at the node indicate the levels of bootstrap support based on 1000 resampled datasets. The scale bar represents changes per nucleotide. (**a**) Phylogenetic tree of *Streptomyces* spp.; (**b**) Non-*Streptomyces* Actinobacteria; (**c**) Bacteria other than Actinobacteria. Species in bold-face type represent the closest active strains and the rest are sequence from databases.

About 58% of genera in the phylum Actinobacteria showed antimicrobial activity compared to 10% of the activity from the other genera. Eighty percent of members of the genus *Streptomyces* showed antimicrobial activity. In contrast, the activity for more than half of the other tested genera was not detected under the experimental conditions used. The phylogenetic tree of the active strains is displayed in Figure 2. Sequence lengths of 1200–1300 Bp were used to generate the tree. Strains from this study represent in bold taking the species name of the closest strain from BLAST analysis.

### 3.4. Role of Media for Antimicrobial Production

To investigate the influence of primary production media on antimicrobial production, eight pre-screened highly active bacterial strains were sub-cultured in five media and tested against *S. aureus,* including MRSA. As shown in Table 3, a marked variation in antimicrobial production by specific strains depends on the medium used. International *Streptomyces* broth medium #2 (ISP2) promoted a certain degree of antimicrobial production by all strains, though the number of strains producing antimicrobials in mannitol soybean (MS) medium was lower compared to ISP2, they produced larger zones of inhibition. Yeast extract peptone (YP) did not support antimicrobial production while there was negligible production in starch yeast extract peptone (SYP) medium (Table 3).

The next phase was to scale-up production in liquid production medium. To achieve this the three most active strains (RB27, RB53, and RB154) were grown in four liquid production media. In general, good antimicrobial production was detected after three days of incubation and maximum activity observed by day seven. Specifically, MS production medium promoted better production with larger zones of inhibition (Table 4) for two of the three cultures.

### 3.5. Antimicrobial Production from Strain RB27, Preliminary Investigation and Purification

Strain RB27 grown in MS medium was selected for further study as it showed high activity versus both *S. aureus* and MRSA. Several batches of shake flasks were setup for seven-day fermentations to collect a total of five liters of fermentation broth from RB27. Flasks which showed antibiotic activity on day 7 were centrifuged and supernatant pooled for further purification.

Preliminary investigation of the RB27 extract revealed that the active compound was not extracted with butanol, chloroform, or ethyl acetate as the solvents only remove portions of the broth that did not show any inhibitory activities. Furthermore, the active extract was completely soluble in water but not in methanol. It was also discovered that the RB27 extract maintained its activity at room temperature for more than a month. The water-soluble antibiotic was therefore purified by sequentially removing impurities from the crude extract as opposed to the usual purification protocols where the active compound is removed from crude extracts. The purified compound was tested for activity and showed strong inhibitory activity.

### 3.6. Bioautogram of the Purified Compounds and Their Identification

The bioautogram of the purified compound from strain RB27 was determined using silica gel 60 F_254_ thin layer chromatography (TLC) (Merck 5554 aluminium sheets) with different solvent systems. The result indicated that only the solvent system consisting of pyridine: 6MHCl: RO water: methanol (10:4:26:80) was able to elute the compound, as only this elution solvent showed an antimicrobial zone of inhibition.

The purified compound was analysed by nuclear magnetic resonance spectroscopy (NMR) and mass spectrometry. The 1 and 2-dimensional ^1^H-NMR spectra are shown in Supplementary Figure S1. These ^1^H spectra of the RB27 extract were checked for their identity against the reference spectra in available databases revealing no match, indicating the possibility that it has a novel chemical structure. The accurate mass spectra (Supplementary Figure S2) showed common contaminant peaks except for 685.4340. The potential formula for this peak was assessed and the most related formulae are indicated in Supplementary Table S3.

### 3.7. Minimum Inhibitory Concentration and Mnimum Bactericidal Concentration of the Compound

As indicated in Table 5, the purified compound was tested against 15 pathogenic bacteria. This resulted in a minimum inhibitory concentration (MIC) (mg/mL) of 0.3125 to 1.25 and minimum bactericidal concentration (MBC) (mg/mL) of 1.25 to 2.5 for MRSA, *S. aureus, E. faecalis, C. striatum*, *S.* *agalactiae,* and *S.* *capitis.*

## 4. Discussion

The marine environments of South Australia contain large numbers of sponge species, with the majority being endemic. In this study, the bacterial communities of twelve sponge samples were investigated by culturing to obtain a total of 1234 isolates with different morphological forms. In comparison, similar studies from Brazil with a similar number of sponge samples yielded a much lower number of 158 [62]. Previous studies have reported a reasonable amount of bacteria from marine sponges with differing numbers and morphological forms [63,64,65]. Genotypic analysis revealed the isolates belong to four phyla, Actinobacteria, Firmicutes, Proteobacteria, and Bacteroidetes comprised of 23 genera. In terms of phyla diversity, the same types and number of phyla have been reported previously [32,63,64,66,67]. The relative abundance of each phylum varies substantially where phylum Actinobacteria was the most abundant followed by Firmicutes, Proteobacteria and an insignificant amount of Bacteroidetes. In agreement with our report, a high abundance of phylum Actinobacteria has been found previously [32,68,69]. However, some studies have also reported a low level of the phylum Actinobacteria compared to Proteobacteria [64,66,67,70].

A total of 169 bacterial isolates were screened for antimicrobial activities with 42% displaying activity against bacterial and fungal pathogens. This percentage of overall antimicrobial activity was well within the range found in other comparable studies, irrespective of the number or types of sponge [71,72,73]. This study also reported a higher percentage of overall antimicrobial activities compared to other related studies [56,67,70,74,75]. General antimicrobial activity profiles of 10–65% have been indicated in various studies [32,62,64,66,74], though factors such as the number of strains tested and assay conditions affect the overall antimicrobial production [32].

Specific analysis of antimicrobial profiles varies from study to study. For example, in line with our study, a lack of activity against Gram-negative bacteria has been observed [73], whereas others have seen activity [27,50,62,71,72]. Antifungal activities, on the other hand, were reported by Kuo et al. (2019) [67] but not in other similar studies [72,74]. These variations in the range of antimicrobial activities could be due to the difference of the environment surrounding the sponges which favour the development of specific defence mechanisms over others. As many as 90% of bacteria with antimicrobial activities displayed inhibition towards two or more of the tested pathogens. These findings may indicate that the marine environments, especially marine sponges, could be a unique environment that demands the symbionts to develop multiple forms of survival strategies, including antimicrobial compounds.

Ten of the 21 genera showed antimicrobial activity including *Streptomyces*, *Gordonia*, *Kocuria*, *Pseudonocardia*, *Microbacterium*, *Micrococcus*, *Bacillus*, *Fictibacillus*, *Sulfitobacter*, and *Limimaricola*. The phylogenetic analysis of the active bacterial isolates indicated that most of them belong to phylum Actinobacteria, particularly the genus *Streptomyces*, which are known for their ability to produce various types of biologically active compounds [76]. While this finding was supported by similar studies, a lower percentage of antimicrobial activities by phylum Actinobacteria were reported compared to other phyla [27,64,66,72].

Many of the genera reported in this study have been described as having antimicrobial activities. In addition to *Streptomyces*, antimicrobial activity has also been attributed to *Bacillus* [27,30,32,60,62,64,66,67,69,72], *Micrococcus* [71,72,74], *Microbacterium* [73], *Gordonia* [72], *Sulfitobacter* [32], and *Kocuria* [35]. Antimicrobial activities due to *Fictibacillus* and *Limimaricola* were not reported, though phylogenetically, the genus *Fictibacillus* is highly related to *Bacillus* and *Limimaricola* with *Sulfitobacter*. This observation indicates that the search for new antimicrobials should consider less common and unexplored genera.

An observation of this study is the inconsistent distribution of genera which showed antimicrobial activity. Some genera, such as *Pseudovibrio*, *Pseudoalteromonas*, *Vibrio*, *Bacillus*, and *Proteus,* were the most common antimicrobial-producing isolates [27,64,66,72,74,75,77] but this was not observed in our study. Several factors may contribute to this observation. Firstly, specific genus/genera may dominate in some geographical areas and are thus screened in higher numbers compared to the less common ones. Secondly, the variation could be due to a methodological factor. Here, a similar study which screened only a few representatives from the group could miss the active strain. Thirdly, could be related to the expert effect. Researchers may have a different skill level for identification of one genus over the other and this would lead to the preferential incorporation of one genus in the study over the other. Whether it was a real difference or a personal bias, the observed variations in genera in terms of activities among studies encourages a search for more active genera as alternative sources of antimicrobials.

The importance of appropriate media and incubation time for optimal bioactive compound production was demonstrated in this study and shown to influence the outcome of the screening results. Eight highly active bacterial strains were tested for antimicrobial production using multiple liquid media. The results indicated a marked preference of strains for media to produce bioactive compounds with different degrees of inhibition which is like other related studies [27,66,73]. Media such as ISP2 and MS supported antimicrobial production for most of the tested strains. All these studies recognized the importance of media for the isolation of larger amounts and proportions of bacterial isolates with bioactivities.

This study not only highlighted the importance of media for primary screening but also large-scale liquid state production of bioactive compounds. Here, not only the media but the time of incubation also influenced production. Most antimicrobial activities were observed after three days of fermentation from the three-day old seed medium with the inhibitory activity increasing until day seven. Graça et al. (2013) reported the importance of media and the proper time for antimicrobial production in the liquid state fermentation [72]. A recent review also indicated the importance of medium optimization (pH, temperature, agitation speed, incubation period, medium composition) for optimum yield of fermentation products [78].

This study purified one active water-soluble compound from strain RB27 which showed strong inhibitory activity against *S. aureus*. 16S rRNA sequence analysis of RB27 classified the isolate to belong to the genus *Streptomyces* with 99.83% sequence similarity to *Streptomyces lienomycini* LMG 20091^T^. We used an unusual method of purification by removing impurities in a sequential treatment. This approach resulted in an almost pure compound. Various similar studies [64,77,79] followed different approaches and successfully extract pure compounds with antimicrobial activities.

Preliminary findings, comparing the online database with NMR and accurate mass data, suggest that this compound could be novel. It has a molecular weight of 683 Daltons and possibly contains three to four sugar molecules with OH/NH groups. Activity studies against 15 pathogenic bacteria revealed an MIC of 0.3125 to 1.25 (mg/mL) and MBC of 1.25 to 2.5 (mg/mL) for MRSA*, S. aureus, E. faecalis*, *C. striatum, *S.* agalactiae,* and *S.* *capitis.* This initial observation is encouraging for future work in purifying compounds from additional strains which showed antibacterial and antifungal activities. This result also indicated the possibility of discovering new compounds not only from novel bacteria, but also from known bacteria from marine sponges, as the environment may force the bacteria to produce such bioactive compounds.

## 5. Conclusions and Future Directions

The number and the morphological diversity of bacterial isolates from the study area varies depending on the source of the sponges. Dominancy of actinobacterial genera was observed. Quite a remarkable percentage of the selected isolates displayed antimicrobial activities against *S. aureus,* *C. albicans*, *T. rubrum*, *T. interdigitalis*, *M. gypsum,* and *Sacclophosis* sp. Bacteria under the phylum *Actinobacteria*, particularly genus *Streptomyces,* showed marked antimicrobial activities compared to other isolates in the tested experimental conditions. Antimicrobial production greatly depended on the genus type, medium ingredients, and time of incubation. One new antibiotic compound was purified.

Marine sponges of South Australia contain uncommon bacterial genera possessing antimicrobial activities, encouraging future explorations to identify novel antimicrobials. In this study, only representative isolates were screened for activities and not all the strains were isolated due to capacity limitations. Therefore, future studies should screen more strains and focus on the characterization of potential novel antimicrobials, including testing them for cell toxicity.

## Figures and Tables

**Table 1:** Bacteria isolated from different sponges.

Sample Code	Sample ID	SYP	AP	NS	HV	MA	NA	TS	Total
GB_SP_01	*Geodia* sp.	4 (1) *	5 (2)	-	3 (1)	1 (1)	2 (1)	1 (1)	16 (7)
GB_SP_08	*Chondrosida* sp.	7 (2)	14 (4)	4 (2)	6 (2)	2 (1)	2 (1)	1 (1)	36 (14)
GB_SP_ 21	*Chondrosida* sp.	-	4 (1)	3 (1)	-	3 (1)	3 (1)	2 (1)	15 (5)
GB_SP_23	*Chondrosida* sp.	5 (2)	7 (3)	-	5 (2)	6 (2)	6 (2)	-	29 (11)
RB_SP_01	*Ircinia* sp.	-	8 (3)	-	6 (3)	-	5 (2)	7 (3)	26 (11)
RB_SP_02	*Poecilosclerida* sp.	2 (1)	4 (2)	-	2 (1)	5 (2)	-	-	13 (6)
RB_SP_03	*Crella* sp.	-	-	-	-	4 (2)	4 (2)	4 (2)	12 (6)
RB_SP_11	*Sarcotragus* sp.	6 (3)	4 (2)	-	3 (1)	5 (2)	2 (1)	-	20 (9)
RB_SP_12	*Carteriospongia foliascens*	-	4 (2)	-	3 (1)	-	-	4 (2)	11 (5)
RB_SP_16	*Aplysilla sulfurea*	17 (8)	35 (16)	6 (3)	28 (14)	11 (5)	12 (6)	8 (4)	117 (56)
RB_SP_17	*Dendrilla* sp.	3 (1)	10 (4)	-	5 (2)	6 (3)	4 (2)	2 (1)	30 (13)
RB_SP_18	*Tedania tubulifera*	9 (4)	15 (6)	2 (1)	10 (5)	7 (3)	7 (3)	8 (4)	58 (26)

* total number of unique morphological and microscopic forms of bacterial isolates from each sponge sample and the number of representative bacterial isolates selected for screening shown in ().—represents bacterial isolates not observed in isolation medium. SYP: Starch yeast peptone agar; AP: Asparagine peptone agar; NS: Natural seawater agar; HV: Humic acid vitamin agar; MA: Marine agar; NA: Nutrient agar; TS: Tryptone soya agar.

**Table 2:** Antimicrobial activities of 169 bacterial strains.

Strains	Phylum	Positive *	Negative	Total Screened
*Streptomyces*	Actinobacteria	58	14	72
*Gordonia*	Actinobacteria	1	2	3
*Bacillus*	Firmicutes	3	23	26
*Fictibacillus*	Firmicutes	1	4	5
*Sulfitobacter*	Proteobacteria	1	7	8
*Kocuria*	Actinobacteria	2	7	9
*Pseudonocardia*	Actinobacteria	1	1	2
*Microbacterium*	Actinobacteria	1	6	7
*Limimaricola*	Proteobacteria	1	3	4
*Janibacter*	Actinobacteria	0	1	1
*Muricauda*	Bacteroidetes	0	1	1
*Staphylococcus*	Firmicutes	0	2	2
*Micrococcus*	Actinobacteria	1	5	6
*Falsibacillus*	Firmicutes	0	5	5
*Rhodovulum*	Proteobacteria	0	2	2
*Mycolicibacterium*	Actinobacteria	0	1	1
*Rhodococcus*	Actinobacteria	0	8	8
*Pseudomonas*	Proteobacteria	0	3	3
*Leisingera*	Proteobacteria	0	1	1
*Isoptericola*	Actinobacteria	0	2	2
*Pseudoalteromonas*	Proteobacteria	0	1	1
Total		70	99	169

* Positive (showed inhibitory activities); Negative (No inhibition).

**Table 3:** Antimicrobial production of selected bacterial strains using different liquid media.

Strains	YP *	PD	SYP	MS	ISP2
RB27	-	14	-	26	26
RB53	-	16	-	29	19
RB154	-	-	10	21	26
RBYA1	-	-	14	18	16
RBYA2	-	14	-	20	18
RBYA21	-	-	14	-	21
RBYA22	-	-	-	-	16
RBYA32	-	14	-	18	22

* YP: Yeast extract peptone; PD: Potato dextrose; SYP: Starch yeast extract peptone, MS: Mannitol soybean; ISP2: International *Streptomyces* medium. The number indicates the zone of inhibition (mm) against *S. aureus*.

**Table 4:** Antimicrobial production of three selected active strains in four liquid fermentation media.

Strains	Media	Extract													
		Day 1		Day 2		Day 3		Day 4		Day 5		Day 6		Day 7	
		SA	MRSA	SA	MRSA	SA	MRSA	SA	MRSA	SA	MRSA	SA	MRSA	SA	MRSA
**RB27**	ISP2	-	-	23	19	27	23	23	20	24	21	23	20	26	19
	SM	-	-	14	-	12	-	14	13	17	15	15	-	-	-
	MS	-	-	27	26	27	27	28	29	28	28	28	26	29	28
	GM	-	-	21	15	21	17	23	18	23	17	23	20	24	19
**RB53**	ISP2	-	-	16	-	17	-	14	-	-	-	-	-	7	-
	SM	-	-	-	-	-	-	-	-	-	-	-	-	-	-
	MS	-	-	-	-	22	19	22	18	22	21	22	19	22	19
	GM	-	-	-	-	-	-	-	-	-	-	-	-	-	-
**RB154**	ISP2	13	13	13	13	16	14	16	11	11	8	9	8	18	10
	SM	14	17	17	18	17	19	13	19	13	13	13	16	13	13
	MS	14	14	15	17	13	17	12	12	13	17	-	-	-	-
	GM	16	17	18	20	18	19	19	18	17	19	18	20	18	20

-Zone of inhibition in mm. SM-sucrose medium; GM-glucose medium.

**Table 5:** MIC and MBC values of the active compound from RB27 strain against 16 types of bacteria.

Strains	MIC (mg/mL)	MBC (mg/mL)
Methicillin-resistant *Staphylococcus aureus*	1.25	2.5
*Staphylococcus aureus*	0.625	1.25
*Enterococcus faecalis*	0.625	1.25
*Corynebacterium striatum*	1.25	2.5
*Streptococcus* *agalactiae*	1.25	2.5
*Staphylococcus* *capitis*	0.3125	1.25
*Klebsiella pneumoniae*	>10	>10
*Corynebacterium* sp.	>10	>10
*Pseudomonas aeruginosa*	>10	>10
*Escherichia coli*	>10	>10
*Micrococcus* sp.	>10	>10
*Bacillus cereus*	>10	>10
*Staphylococcus epidermidis*	>10	>10
*Fictibacillus arsenicus*	>10	>10
*Stenotrophomonas maltophilia*	>10	>10
*Streptococcus pyogenes*	>10	>10

## Data Availability

The accession numbers for the genome sequences were provided in the text.

## Supplementary Materials

The following are available online at https://www.mdpi.com/2076-2607/9/1/171/s1, Supplementary Table S1. 169 bacterial isolates screened for antimicrobial activities. Supplementary Table S2. Bacterial isolates with antimicrobial activities against at least one of the test organisms. Supplementary Table S3. Possible formula of a compound from RB27. Supplementary Figure S1. ^1^H-NMR spectra of extract from strain RB27 in DMSO-d6. Supplementary Figure S2. Mass spectra of extract from strain RB27 using HDMS*^TOF MS ES+^*.
