# Supplementary material for: Antimicrobial Activities of Marine Sponge-Associated Bacteria
Source: Microorganisms. 2021 Jan 14;9(1):171. doi: 10.3390/microorganisms9010171 (PMC7830929; doi:10.3390/microorganisms9010171)
Supplement: Supplementary file 1 [file microorganisms-09-00171-s001.zip › Supplementary Figure S1 NOV 10.docx]

Supplementary Figure S1. ^1^H-NMR spectra of extract from strain RB27 in DMSO-d6. **a** 1 dimensional 1H spectrum; **b** expanded 1 dimensional 1H spectrum; **c & d** 2D correlation spectroscopy (COSY) (^1^H, ^1^H)-NMR spectrum where the horizontal axis is defined as F2 (direct dimension) and the vertical axis as F1 (indirect dimension).
