# Supplementary material for: Antimicrobial Activities of Marine Sponge-Associated Bacteria
Source: Microorganisms. 2021 Jan 14;9(1):171. doi: 10.3390/microorganisms9010171 (PMC7830929; doi:10.3390/microorganisms9010171)
Supplement: Supplementary file 1 [file microorganisms-09-00171-s001.zip › Supplementary Figure S2 Nov 10.docx]

Supplementary Figure S2. Mass spectra of high definition mass spectrometer (HDMS)^TOF MS ES+^. **a** mass scan of the extract from 100 to 800 Daltons; **b** zooms of mass scan 650 to 800 Daltons. The arrow points the peak of the compound of interest as none of the internal contaminant peaks much with it.
