# Supplementary material for: Antimicrobial Activities of Marine Sponge-Associated Bacteria
Source: Microorganisms. 2021 Jan 14;9(1):171. doi: 10.3390/microorganisms9010171 (PMC7830929; doi:10.3390/microorganisms9010171)
Supplement: Supplementary file 1 [file microorganisms-09-00171-s001.zip › Supplementary Table S1 Nov10.docx]

Supplementary Table S1. 169 bacterial isolates screened for antimicrobial activities

| Genus | Strain name | RFLP groups | Strain name | RFLP groups | Strain name | RFLP groups | Strain name | RFLP groups |
| --- | --- | --- | --- | --- | --- | --- | --- | --- |
| *Streptomyces* (n=72) | RB19 | 31 | RB116 | 31 | RBYA7 | 31 | RB26 | 32 |
|  | RB29 | 31 | RB117 | 31 | RBYA8 | 31 | RB27 | 32 |
|  | RB31 | 31 | RB119 | 31 | RBYA13 | 31 | RB28 | 32 |
|  | RB45 | 31 | RB124 | 31 | RBYA20 | 31 | RB30 | 32 |
|  | RB46 | 31 | RB134 | 31 | RBYA21 | 31 | RB32 | 32 |
|  | RB49 | 31 | RB135 | 31 | RBYA22 | 31 | RB33 | 32 |
|  | RB53 | 31 | RB140 | 31 | RBYA24 | 31 | RB34 | 32 |
|  | RB57 | 31 | RB145 | 31 | RBYA27 | 31 | RB35 | 32 |
|  | RB60 | 31 | RB146 | 31 | RBYA28 | 31 | RB36 | 32 |
|  | RB65 | 31 | RB147 | 31 | RBYA32 | 31 | RB54 | 32 |
|  | RB66 | 31 | RB150 | 31 | RBLC14 | 37 | RB111 | 32 |
|  | RB67 | 31 | RB151 | 31 | Rb47 | 38 | RB124 | 32 |
|  | RB69 | 31 | RB152 | 31 | RB15 | 32 | RB131 | 32 |
|  | RB70 | 31 | RB154 | 31 | RB20 | 32 | RB144 | 32 |
|  | RB74 | 31 | RB155 | 31 | RB21 | 32 | RB243 | 32 |
|  | RB76 | 31 | RB158 | 31 | RB22 | 32 |  |  |
|  | RB112 | 31 | RBYA1 | 31 | RB23 | 32 |  |  |
|  | RB114 | 31 | RBYA2 | 31 | RB24 | 32 |  |  |
|  | RB115 | 31 | RBYA3 | 31 | RB25 | 32 |  |  |
| *Bacillus* (n=26) | GB21 | 1 | RB199 | 5 | GB66 | 5 | RBL30 | 5 |
|  | RB1 | 7 | RB13 | 7 | RB90 | 7 | RB92 | 7 |
|  | RB98 | 7 | RB99 | 7 | RB141 | 7 | RB160 | 7 |
|  | RB178 | 7 | RB193 | 7 | GB2 | 7 | GB25 | 7 |
|  | GB48 | 7 | GB52 | 7 | GBYA14 | 7 | GBYA15 | 7 |
|  | GBYA24 | 7 | RBLC2 | 7 | RB97 | 13 | GB14 | 13 |
|  | RBYA37 | 35 | GB3 | 36 |  |  |  |  |

Supplementary Table S1 (Continued)

| Genus | Strain name | RFLP groups | Strain name | RFLP groups | Strain name | RFLP groups | Strain name | RFLP groups |
| --- | --- | --- | --- | --- | --- | --- | --- | --- |
| *Kocuria* (n=9) | RB9 | 21 | RB10 | 21 | RB11 | 21 | RB16 | 21 |
|  | RB44 | 21 | RBYA2 | 31 | RB17 | 33 | RB100 | 33 |
|  | RB107 | 34 |  |  |  |  |  |  |
| *Sulfitobacter*  (n=8) | GB40 | 2 | GB42 | 2 | GB49 | 2 | RB58 | 2 |
|  | GB6 | 26 | GB26 | 27 | GB39 | 27 | GB20 | 28 |
| *Rhodococcus* (n=8) | GB24 | 12 | GB63 | 12 | RB163 | 12 | RBYAN | 12 |
|  | RBLC21 | 12 | RB75 | 12 | RB82 | 12 | RBYA12 | 12 |
| *Microbacterium* (n=7) | RB197 | 20 | RB206 | 20 | GB56 | 20 | RBA4 | 20 |
|  | RB4 | 20 | RB4 | 20 | RB2 | 20 |  |  |
| *Micrococcus*  (n=6) | RB5 | 23 | RBLC12 | 24 | RBYA34 | 26 | RBYA39 | 26 |
|  | RB166 | 29 | GB9 | 29 |  |  |  |  |
| *Flasibacillus* (n=5) | RB184 | 10 | GB74 | 10 | GB75 | 10 | GBYA47 | 10 |
|  | RBLC16 | 10 |  |  |  |  |  |  |
| *Fictibacillus* (n=5) | RB189 | 9 | GB73 | 9 | GB72 | 9 | RBYAC | 9 |
|  | RBLC7 | 9 |  |  |  |  |  |  |
| *Limimaricola* (n=4) | RB6 | 6 | GB11 | 4 | GB45 | 6 | GB69 | 6 |
| *Gordonia* (n=3) | RB180 | 15 | RB194 | 15 | GB17 | 15 |  |  |
| *Pseudomonas* (n=3) | RB161 | 14 | RB198 | 14 | GB4 | 14 |  |  |
| *Isoptericola* (n=2) | RB202 | 19 | RBL40 | 19 |  |  |  |  |

Supplementary Table S1 (Continued)

| Genus | Strain name | RFLP groups | Strain name | RFLP groups | Strain name | RFLP groups | Strain name | RFLP groups |
| --- | --- | --- | --- | --- | --- | --- | --- | --- |
| *Pseudonocardia* (n=2) | RB78 | 30 | RBYA11 | 30 |  |  |  |  |
| *Rhodovulum* (n=2) | GB76 | 11 | GB79 | 11 |  |  |  |  |
| *Staphylococcus* (n=2) | RB176 | 18 | RBL11 | 18 |  |  |  |  |
| *Janibacter* (n=1) | GB23 | 1 |  |  |  |  |  |  |
| *Leisingera* (n=1) | RB86 | 16 |  |  |  |  |  |  |
| *Muricauda* (n=1) | GB37 | 3 |  |  |  |  |  |  |
| *Mycolicibacterium* (n=1) | RBLC1 | 17 |  |  |  |  |  |  |
| *Pseudoalteromonas* (n=1) | RB122 | 25 |  |  |  |  |  |  |

A total of 38 RFLP groups were generated with restriction enzymes which comprised of 383 bacterial strains. From each RFLP group, representative bacterial isolates were selected to test for antimicrobial activity. For some RFLP groups, all bacterial isolates were screened due to their low numbers. Many bacterial isolates from the genus *Streptomyces* were included as this genus was the most dominant isolated in this study.
