# Supplementary material for: Antimicrobial Activities of Marine Sponge-Associated Bacteria
Source: Microorganisms. 2021 Jan 14;9(1):171. doi: 10.3390/microorganisms9010171 (PMC7830929; doi:10.3390/microorganisms9010171)
Supplement: Supplementary file 1 [file microorganisms-09-00171-s001.zip › Supplementary Table S2 Nov 10.docx]

Supplementary Table S2. Bacterial isolates with antimicrobial activities

| Strains name | RFLP group | genus | *S. aureus* (SF) | *S. aureus* (LF) | MRSA (SF) | MRSA (LF) | *C. albicans* (SF) | *C. albicans* (LF) | *T. rubrum* | *T. interdigitalis* | *Sacclophosis sp.* | *M. gypsum* |
| --- | --- | --- | --- | --- | --- | --- | --- | --- | --- | --- | --- | --- |
| RB28 | 32 | *Streptomyces* | 19 | 21 | 16 | 18 | - | - | - | - | +3 | +3 |
| RB30 | 32 | *Streptomyces* | 16 | 19 | 16 | 19 | - | - | - | - | - | - |
| RB34 | 32 | *Streptomyces* | 17 | 20 | - | - | 17 | 19 | +2 | +2 | +2 | +4 |
| RB35 | 32 | *Streptomyces* | 18 | 20 | 17 | 20 | - | - | - | - | - | - |
| RB36 | 32 | *Streptomyces* | 16 | 16 | 17 | 20 | - | - | - | +2 | +2 | - |
| RB45 | 31 | *Streptomyces* | 21 | 23 | 18 | 19 | - | - | +1 | +2 | +2 | +2 |
| RB46 | 31 | *Streptomyces* | 16 | 20 | - | - | 17 | 21 | +3 | +2 | +3 | - |
| RB49 | 31 | *Streptomyces* | 17 | 21 | 17 | 19 | - | - | - | - | - | - |
| RB53 | 31 | *Streptomyces* | 19 | 22 | 18 | 21 | - | - | - | - | - | - |
| RB54 | 32 | *Streptomyces* | 18 | 20 | 17 | 19 | 17 | 20 | +2 | +3 | - | - |
| RB57 | 31 | *Streptomyces* | 16 | 19 | 16 | 18 | - | - | - | - | - | - |
| RB60 | 31 | *Streptomyces* | 18 | 21 | 17 | 20 | - | - | - | - | - | - |
| RB65 | 31 | *Streptomyces* | 16 | 19 | 17 | 22 | - | - | - | - | - | - |
| RB66 | 31 | *Streptomyces* | 17 | 22 | - | - | 16 | 18 | +3 | +3 | +1 | +3 |
| RB67 | 31 | *Streptomyces* | 17 | 21 | - | - | 16 | 18 | +3 | +3 | +3 | +3 |
| RB70 | 31 | *Streptomyces* | 16 | 18 | - | - | - | - | +3 | +1 | +3 | +2 |
| RB74 | 31 | *Streptomyces* | 17 | 21 | - | - | 17 | 20 | +2 | +1 | - | - |
| RB76 | 31 | *Streptomyces* | 18 | 22 | 17 | 22 | - | - | - | - | - | - |
| RBYA1 | 31 | *Streptomyces* | 18 | 22 | 18 | 21 | 18 | 20 | +3 | +3 | +3 | +3 |
| RBYA13 | 31 | *Streptomyces* | 18 | 21 | 18 | 18 | 16 | 19 | +3 | +3 | +3 | +2 |
| RBYA18 | 31 | *Streptomyces* | - | - | - |  | 17 | 19 | - | - | - | - |
| RBYA2 | 31 | *Streptomyces* | 18 | 21 | 18 | 20 | - | - | - | - | +1 | +1 |
| RBYA20 | 31 | *Streptomyces* | 16 | 21 | - | 16 | 18 | 22 | +4 | +4 | +4 | +4 |
| RBYA21 | 31 | *Streptomyces* | 19 | 21 | 19 | 21 | 17 | 20 | +3 | +3 | +3 | +2 |
| RBYA22 | 31 | *Streptomyces* | 18 | 18 | 19 | 21 | - | - | +2 | +2 | +1 | +2 |
| RBYA27 | 31 | *Streptomyces* | 16 | 18 | 16 | 17 | 18 | 21 | - | - | - | - |
| RBYA28 | 31 | *Streptomyces* | 18 | 24 | 18 | -20 | - | - | +4 | +4 | +4 | +4 |
| RBYA3 | 31 | *Streptomyces* | 16 | 19 | 18 | 25 | 19 | 26 | +4 | +3 | +2 | +4 |
| RBYA32 | 31 | *Streptomyces* | 18 | 22 | 18 | 19 | 16 | 19 | +4 | +3 | +2 | +3 |
| RBYA7 | 31 | *Streptomyces* | - | - | - | - | 16 | 18 | +3 | +3 | +3 | +3 |
| RBYA8 | 31 | *Streptomyces* | - | - | - | - | 16 | 18 | - | - | - | - |
| RB58 | 2 | *Sulfitobacter* | 17 | 20 | - | - | 17 | 19 | - | - | - | - |
| RB115 | 31 | *Streptomyces* | 17 | 20 | - | - | 16 | 18 | +2 | +1 | - | - |
| RB118 | 32 | *Streptomyces* | 18 | 19 | - | - | 16 | 19 | +2 | - | - | - |
| RB119 | 31 | *Streptomyces* | 17 | 20 | 16 | 18 | - | - | - | - | - | - |

Supplementary Table S2 (Continued)

| Strains name | RFLP group | genus | *S. aureus* (SF) | *S. aureus* (LF) | MRSA (SF) | MRSA (LF) | *C. albicans* (SF) | *C. albicans* (LF) | *T. rubrum* | *T. interdigitalis* | *Sacclophosis sp.* | *M. gypsum* |
| --- | --- | --- | --- | --- | --- | --- | --- | --- | --- | --- | --- | --- |
| RB131 | 32 | *Streptomyces* | 20 | 22 | 17 | 18 | 19 | 21 | +4 | +1 | +3 | +3 |
| RB134 | 31 | *Streptomyces* | 16 | 21 | 16 | 18 | - | - | - | - | - | - |
| RB135 | 31 | *Streptomyces* | 16 | 16 | - | - | - | - | - | - | +3 | - |
| GB48 | 7 | *Bacillus* | 16 | 19 | - | - | 17 | 21 | - | +3 | +1 | - |
| RBL30 | 7 | *Bacillus* | 18 | 21 | - | - | 16 | 18 | - | +2 | - | - |
| GBYA15 | 7 | *Bacillus* | 16 | 20 | - |  | - | - | - | - | +2 | +1 |
| RB98 | 7 | *Bacillus* | - | - | - | - | - | - | - | - | +2 | +1 |
| RBLC7 | 9 | *Fictibacillus* | - | - | - |  | 19 | 21 | - | +2 | - | - |
| GB17 | 15 | *Gordonia* | 16 | 19 | 16 | 18 | - | - | - | - | - | - |
| RB71 | 33 | *Kocuria* | 19 | 21 | - | - | 16 | 20 | - | - | - | +2 |
| RB16 | 21 | *Kocuria* | 19 | 19 | - | - | - | - | - | - | - | - |
| RBYA34 | 26 | *Micrococcus* | 16 | 19 | - |  | 16 | 18 | - | - | +2 | - |
| RB2 | 20 | *Microbacterium* | - | - | - | - | 18 | - | - | - | +3 | - |
| RBYA11 | 30 | *Pseudonocardia* | 18 | 20 | - |  | 16 | 18 | +1 | - | - | +2 |
| RB144 | 32 | *Streptomyces* | - | 18 | 22 | - | 18 | 20 | +2 | +2 | +2 | +3 |
| RB145 | 31 | *Streptomyces* | - | 18 | 21 | 18 | 23 | - | - | +1 | +2 | +1 |
| RB146 | 31 | *Streptomyces* | - | 19 | 21 | - | 17 | 20 | - | +3 | +3 | +4 |
| RB147 | 31 | *Streptomyces* | - | 16 | 18 | 17 | 19 | 16 | - | +3 | +2 | - |
| RB15 | 32 | *Streptomyces* | - | 18 | 20 | - | - | - | - | - | - | +2 |
| RB150 | 31 | *Streptomyces* | - | 17 | 20 | - | 17 | 20 | - | - | - | +2 |
| RB151 | 31 | *Streptomyces* | - | - | - | - | 18 | 21 | - | +2 | +2 | +1 |
| RB152 | 31 | *Streptomyces* | - | 17 | 20 | - | - | - |  | - | - | - |
| RB154 | 31 | *Streptomyces* | - | 18 | 26 | 16 | 18 | 19 | - | +3 | +3 | +2 |
| RB155 | 31 | *Streptomyces* | - | - | - | - | 16 | 20 | - | - | - | - |
| RB158 | 31 | *Streptomyces* | 16 | 20 | - | - | - | -- | - | - | - | - |
| RB19 | 31 | *Streptomyces* | 18 | 18 | 18 | 19 | - | - | +2 | +3 | - | - |
| RB21 | 32 | *Streptomyces* | 18 | 26 | 14 | 18 | 16 | 20 | - | - | - | - |
| RB22 | 32 | *Streptomyces* | 16 | 19 | 16 | 20 | - | - | - | - | - | +2 |
| RB23 | 32 | *Streptomyces* | 18 | 21 | 16 | 22 | - | - | - | - | - | - |
| RB25 | 32 | *Streptomyces* | 16 | 18 | 18 | 21 | - | - | - | - | - | - |
| RB26 | 32 | *Streptomyces* | 18 | 21 | - | - | 18 | 21 | - | - | - | - |
| RB27 | 32 | *Streptomyces* | 21 | 26 | 21 | 24 | - | - | - | - | - | - |
| RB112 | 31 | *Streptomyces* | 18 | 21 | - | - | - | - | - | +2 | - | - |
| RB47 | 38 | *Streptomyces* | 16 | 16 | - | - | - | - | - | - | - | - |
| RB114 | 31 | *Streptomyces* | 17 | 20 | - | - | - | - | - | - | - | - |

The antimicrobial profile of 70 strains which showed inhibitory activities against resistant and non-resistant strains of *S. aureus* and five fungal strains as presented as a zone of inhibition (mm). All the tested bacteria isolates did not show any activities against *S. typhimurium*, *P. aeruginosa*, *E. coli*, and *S. pyogenes* and the data are not presented in the table. For two strains of *S. aureus and C. albicans antimicrobials from* SF: Solid fermentation and; LF: Liquid fermentation were tested. + indicated the degree of the zone of inhibition as described in Section 5.2.2.3. (-) indicated absence of activities.
