# Supplementary material for: Antimicrobial Activities of Marine Sponge-Associated Bacteria
Source: Microorganisms. 2021 Jan 14;9(1):171. doi: 10.3390/microorganisms9010171 (PMC7830929; doi:10.3390/microorganisms9010171)
Supplement: Supplementary file 1 [file microorganisms-09-00171-s001.zip › Supplementary Table S3 Nov 10.docx]

Supplementary Table S3. Possible formula of a compound from RB27

| Mass | Calc.  Mass |  | mDa | PPM | DBE | i-FIT | i-FIT (Norm) | Formula |
| --- | --- | --- | --- | --- | --- | --- | --- | --- |
| 685.434 | 685.4339 |  | 0.1 | 0.1 | 1.5 | 39.3 | 7.2 | C31 H66 N4 O8 P S |
|  | 685.4338 |  | 0.2 | 0.3 | 14.5 | 42.3 | 10.2 | C42 H61 N4 S2 |
|  | 685.4342 |  | -0.2 | -0.3 | 20.5 | 34.4 | 2.2 | C42 H53 N8 O |
|  | 685.4343 |  | -0.3 | -0.4 | 0.5 | 38.3 | 6.1 | C33 H73 N2 O2 P4 S |
|  | 685.4337 |  | 0.3 | 0.4 | 9.5 | 36.3 | 4.1 | C41 H67 O2 P2 S |
|  | 685.4337 |  | 0.3 | 0.4 | -1.5 | 41.3 | 9.1 | C23 H62 N10 O11 P |
|  | 685.4344 |  | -0.4 | -0.6 | 5.5 | 41.6 | 9.5 | C34 H67 N6 P2 S2 |
|  | 685.4336 |  | 0.4 | 0.6 | 11.5 | 36.6 | 4.4 | C34 H57 N10 O3 S |
|  | 685.4335 |  | 0.5 | 0.7 | 6.5 | 38.2 | 6.1 | C33 H63 N6 O5 P2 |
|  | 685.4346 |  | -0.6 | -0.9 | 10.5 | 34.2 | 2.1 | C39 H62 N2 O6 P |

* Single Mass Analysis of the extract with search elements of C: 0-100 H: 0-1000 N: 0-10 O: 0-50 P: 0-10 S: 0-2. A total of 43785 formula(e) were evaluated with 48 results within limits (all results, up to 1000, for each mass) with Tolerance = 5.0 PPM / DBE: min = -1.5, max = 50.0 and number of isotope peaks used for i-FIT = 3.
